# Early Diagnosis of Nonaneurysmal Infectious Thoracic Aortitis Using Transesophageal Echocardiogram in a Patient with Purulent Meningitis

**DOI:** 10.4061/2009/769694

**Published:** 2010-02-22

**Authors:** Ricardo Lopes, Jorge Almeida, Paula Dias, Paulo Pinho, Maria Júlia Maciel

**Affiliations:** ^1^Serviço de Cardiologia, Hospital São João, Alameda Prof. Hernâni Monteiro, 4200–319 Porto, Portugal; ^2^Serviço de Cirurgia Torácica, Hospital São João, Alameda Prof. Hernâni Monteiro, 4200–319 Porto, Portugal

## Abstract

Infectious thoracic aortitis is a rare entity in the antibiotic era and usually appears in patients with prior aortic disease and/or associated infective endocarditis. Infected nonaneurysmal aorta will likely progress to mycotic aneurysm if left untreated. In most of the reports, infectious thoracic aortitis presents with a mycotic aneurysm. We report the case of a patient with a nonaneurysmal infectious thoracic aortitis, probably secondary to purulent meningitis, early diagnosed by transesophageal echocardiogram.

## 1. Case Report

A 57-year-old man came to our emergency department complaining of headache, asthenia, and high fever (39°C) for 48 hours. He had a liquor fistula and epilepsy as a result of brain trauma at the age of 22, and had already been submitted to several surgical procedures for fistula correction. Furthermore, he had had 2 admissions due to purulent meningitis and was under medication with carbamazepine (400 mg tid) and lorazepam (1 mg/day). Patient's vital signs were: *T* = 38.3°C, BP = 117/76 mmHg, HR = 105 bpm, and RR = 22 cpm. Cardiac examination and pulmonary auscultation were normal. Patient was diaphoretic and with neck rigidity. Blood analysis revealed leukocytosis (17 × 10^12^/L) with neutrophilia (90%) and high C-reactive protein (63 mg/L). The cerebrospinal fluid (CSF) was purulent, with glucose of 54 mg/dL, protein of 0.55 g/L and 2800 cells (80% polymorphonuclear cells). Chest X-ray was normal.

**Figure 1:**
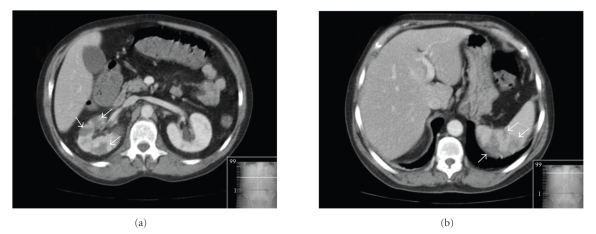
Abdominal computed tomography scan showing multiple ischemic areas (*arrows*) in the right kidney (a) and in the spleen (b), suggesting an embolic source.

**Figure 2:**
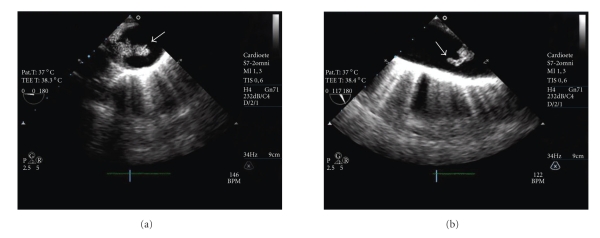
(a) Transesophageal echocardiogram (TEE) image of the proximal descending aorta (PDA) with sectioning plane at 0°. A large polypoid sessile vegetation (2.1 × 0.9 cm) attached to the intima can be seen at the beginning of descending thoracic aorta (*arrow*); (b) TEE image of the PDA with sectioning plane at 117°, showing the same vegetation (*arrow*).

**Figure 3:**
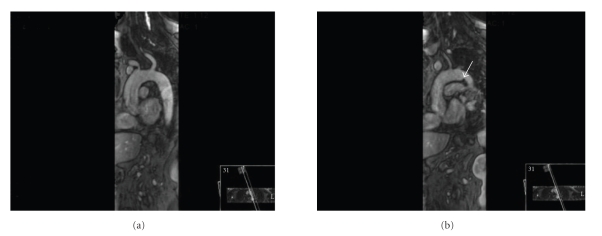
Thoracic MRI performed before surgery, showing normal dimensions of the thoracic aorta (a) and a repletion defect (*arrow*) in the aortic arch-descending thoracic aorta transition compatible with a vegetation (b).

He was admitted into our hospital and started treatment with ceftriaxon (2 g iv bid) with clinical and analytical improvement. On the 11th day on antibiotics he presented high fever (39°C), right lumbar pain, hiccups, and vomiting. Blood analysis showed leukocytosis (30 × 10^12^/L) with neutrophilia (80%) and high C-reactive protein (324 mg/L). Abdominal computed tomography scan revealed multiple ischemic areas in the spleen and in the right kidney (Figures 1(a) and 1(b)), suggesting an embolic source. Transthoracic echocardiogram showed normal cardiac valves and left ventricular function. Transesophageal echocardiogram (TEE) revealed a large (2.1 × 0.9 cm) polypoid sessile vegetation with highly emboligenic characteristics at the beginning of descending thoracic aorta, suggesting infectious thoracic aortitis (Figures 2(a) and 2(b)). The other segments of thoracic aorta appeared normal and there were no signs of infective endocarditis or thrombus in left atrial appendage. He was medicated with intravenous vancomicyn (1 g bid), gentamicyn (80 mg tid), and meropnem (2 g tid) for 4 weeks with clinical and analytical improvement. An additional TEE performed 4 weeks later showed no significant change in vegetation size (1.8 × 0.9 cm) and it was decided to perform surgical treatment (40th day of hospitalization). Thoracic magnetic resonance imaging (MRI) was performed before surgery, showing normal dimensions of the thoracic aorta and a repletion defect in the aortic arch-descending thoracic aorta transition compatible with a vegetation (Figures 3(a) and 3(b)).

**Figure 4:**
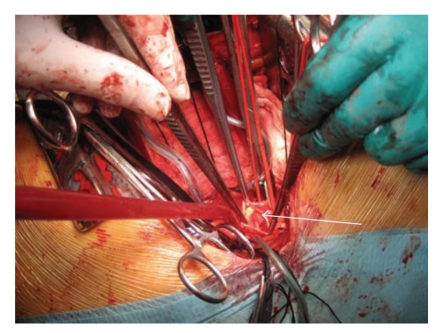
Intraoperative image of the affected thoracic aortic segment with the vegetation (*arrow*).

**Figure 5:**
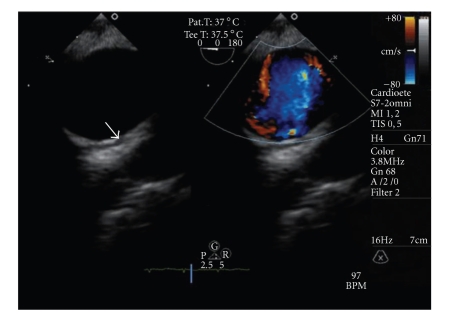
TEE image of the PDA, with sectioning plane at 0°, performed 4 weeks after surgery and before the discharge, showing the aortic conduit (*arrow)*; (left) with a normal colour doppler flow (right).

The affected aortic segment (Figure 4) was ressected with interposition of an aortic conduit (24 mm). Postoperative period was uneventful and antibiotherapy with vancomicyn and meropnem was maintained another 4 weeks. Histopatological examination of the excised segment showed a mild inflammation of the aortic wall and an atheroma plaque with many fibrinous material attached. No etiologic agent was isolated in blood, CSF, and tissue cultures. TEE performed before hospital discharge showed no changes besides the presence of the aortic conduit (Figure 5). The patient had left the hospital 68 days after the admission, medicated with lorazepam (1 mg/day) and carbamazepine (400 mg tid). There were no events to register in the 18 months of follow-up.

## 2. Discussion

Infectious thoracic aortitis (IA) is a rare entity in the antibiotic era and usually appears in patients with prior aortic disease and/or associated infective endocarditis [1, 2]. IA and mycotic aneurysms (MA) are different presentations of the same disease. If left untreated, an infected non-aneurysmal aorta will likely progress to MA. Most of the reports of IA present themselves with an aneurysm, so it is impossible to determine if the aneurysm was already there before the infection occurred [3]. Moreover, there are only a few reports describing IA in the absence of aneurysm [4–7]. Various microorganisms have been associated with IA, most commonly *Salmonella* and *Staphylococcal* species, along with *Streptococcus pneumonia*. However, in up 25% of cases an etiologic agent is not found, maybe due to pretreatment with antibiotics, the absence of an infectious focus in the aortic lumen, or the presence of anaerobic microorganisms as causative agents of the infection [1, 2]. An early diagnosis of IA is very important because this condition is associated with a high rate of rupture and mortality [1]. However, diagnosis is frequently delayed since clinical manifestations are usually nonspecific. TEE is the diagnostic tool of choice to rule out infective endocarditis (IE). If negative, a prompt examination of the thoracic aorta should be done to look for evidence of aneurysm or vegetations [6, 8]. However CT scan with contrast enhancement is considered by many the initial imaging technique of choice, because with TEE upper ascending aorta and proximal aortic arch locations may be missed when trachea is interposed. Magnetic resonance imaging is emerging as a noninvasive imaging modality of choice for IA as it can further define disease extension and help planning surgical intervention [1, 3]. Intravenous large coverage antibiotherapy (extended for at least 6 to 12 weeks after surgical excision and clearence of blood cultures) in combination with complete surgical excision of the infected aorta seems to be the treatment of choice. Early resection of the infected aortic segment before aneurysm formation or rupture is likely to lead to a better prognosis [1–3]. Recently, endovascular aortic repair (EVAR) has been used in the treatment of infected thoracic aortas with promising results, especially in patients without fever or ruptured aneurysms (both postprocedure and 12-month mortality rates of 10%, which is better than surgical mortality rates) [9].

Our patient presented a rare case of nonaneurysmal IA, probably secondary to purulent meningitis. Diagnosis was made early using TEE (while searching for a cardiac source of embolism), which might explain why a MA was not present. He had a single atherosclerotic plaque that was probably seeded by the microorganism causing the meningitis. Although it was not possible to isolate any etiological agent from cultures, we decided to initiate large coverage antibiotherapy, because patient had clinical and analytical signs suggesting bacterial infection. Four weeks of antibiotherapy were performed prior to surgery to improve local surgical conditions, as has been also performed by others [8]. The excised aortic segment showed only a mild inflammation of the aortic wall and an atheroma plaque with many fibrinous material attached, which means that perhaps medical treatment alone would be sufficient. Nevertheless, surgical resection was undertaken, preventing MA formation with subsequent rupture, based on the evidence that surgery combined with medical treatment has a much better prognosis (survival rate of 75%–100% versus 10% with medical treatment alone) [2, 10]. After surgery, antibiotherapy was not extended behind 4 weeks because the patient persistently had negative clinical and analytical signs of infection. Although this would probably be a good case to perform EVAR, we decided not to do it, because our experience is limited and evidence is not yet enough to consider EVAR as an alternative to surgery.

This case highlights the importance of being aware for IA when looking for embolic sources on the suspicion of IE. It also focuses the role of TEE on rapid assessment of the aorta, which may allow an early diagnosis of this entity. An early detection and antibiotherapy in combination with complete surgical excision of the infected aorta are the main steps for a successful treatment.
